# Biomechanics of Chondrocytes and Chondrons in Healthy Conditions and Osteoarthritis: A Review of the Mechanical Characterisations at the Microscale

**DOI:** 10.3390/biomedicines11071942

**Published:** 2023-07-08

**Authors:** Sofia Pettenuzzo, Alessandro Arduino, Elisa Belluzzi, Assunta Pozzuoli, Chiara Giulia Fontanella, Pietro Ruggieri, Valentina Salomoni, Carmelo Majorana, Alice Berardo

**Affiliations:** 1 Department of Civil, Environmental and Architectural Engineering, University of Padova, 35131 Padova, Italy; 2 Musculoskeletal Pathology and Oncology Laboratory, Department of Surgery, Oncology and Gastroenterology, University of Padova (DiSCOG), Via Giustiniani 3, 35128 Padova, Italy; 3 Orthopedics and Orthopedic Oncology, Department of Surgery, Oncology and Gastroenterology, University of Padova (DiSCOG), 35128 Padova, Italy; 4 Department of Industrial Engineering, University of Padova, 35131 Padova, Italy; 5 Department of Management and Engineering (DTG), Stradella S. Nicola 3, 36100 Vicenza, Italy; 6 Department of Biomedical Sciences, University of Padova, 35131 Padova, Italy

**Keywords:** chondrocyte, chondron, biomechanics, biomechanical behaviour, cartilage, osteoarthritis

## Abstract

Biomechanical studies are expanding across a variety of fields, from biomedicine to biomedical engineering. From the molecular to the system level, mechanical stimuli are crucial regulators of the development of organs and tissues, their growth and related processes such as remodelling, regeneration or disease. When dealing with cell mechanics, various experimental techniques have been developed to analyse the passive response of cells; however, cell variability and the extraction process, complex experimental procedures and different models and assumptions may affect the resulting mechanical properties. For these purposes, this review was aimed at collecting the available literature focused on experimental chondrocyte and chondron biomechanics with direct connection to their biochemical functions and activities, in order to point out important information regarding the planning of an experimental test or a comparison with the available results. In particular, this review highlighted (i) the most common experimental techniques used, (ii) the results and models adopted by different authors, (iii) a critical perspective on features that could affect the results and finally (iv) the quantification of structural and mechanical changes due to a degenerative pathology such as osteoarthritis.

## 1. Introduction

Bioengineering studies have drastically increased in number in the last years, demonstrating the importance of this discipline, which is continuously expanding. A subset of bioengineering is biomechanics, which is focused on the study of both the structure and function of biological systems at multiple levels, from the molecular to the system level, in particular, when subjected to external mechanical stimuli [1]. When dealing with cell biomechanics, it has been proven that mechanical stimuli can continuously regulate cell growth, development, regeneration or disease [2,3,4]. Cells can interpret and respond to mechanical signals, thanks to a process called mechanotransduction in which the cell converts these signals into morphological and biochemical changes [2,4,5]. For example, it is known that different external stimuli or changes in the extracellular and pericellular matrix of a cell can alter gene expression and protein secretion. These biochemical modifications can lead to more permanent changes in cell behaviour causing differentiation, migration and apoptosis depending on the type of mechanosensitive pathways activated by these mechanical stimuli [2]. Thanks to this new relevance, the study of cell mechanics and related properties has expanded in the last decades, from both the experimental and the computational point of view. Among the huge variability of cell types, chondrocytes differentiate from mesenchymal cells and present remarkable properties and capabilities. They are the architectural basis of cartilage, a tissue in charge of load bearing, shock absorption and the lubrication of joints throughout the body; they also form the first temporary template for the skeleton during human growth [6]. Chondrocytes are surrounded by the pericellular matrix (PCM) and the two together are referred to as the chondron. In 1925, Benninghoff et al. used the term chondron to identify a structural unit including the chondrocyte and the surrounding microenvironment in hyaline cartilage [7]. The PCM is considered to be a buffer for physical forces between the chondrocyte and the extracellular matrix (ECM) [8]. If chondrocytes are subjected to abnormal mechanical stimuli (e.g., excessive loading, joint trauma or malalignment), their metabolism balance becomes altered, causing matrix loss and tissue degeneration, which can lead to osteoarthritis (OA) [9,10]. Indeed, mechanical stimuli have been recognised among the key factors in the initiation and progression of OA [11]. In this scenario, chondrocyte mechanical properties have become of primary interest to create reliable computational models [12], as well as to better understand the interaction with the PCM and ECM, especially due to the limited ability of articular cartilage (AC) to self-repair [13] and the lack of available clinical treatments to completely repair the tissue. Due to the increasing importance of chondrocyte biomechanics and their correlation with OA, the aim of this review is to analyse the experimental procedures and models developed through the years to study the mechanical behaviour of this cell. In particular, attention has also been paid to those procedures/variables that could have affected the obtained results, such as the isolation procedure used to obtain both chondrocytes and chondrons, the protocol for storing the samples, the type of mechanical tests performed on the samples and the mathematical models adopted to evaluate the results of the experiments performed.

## 2. The Chondrocyte and the Chondron

**Figure 1:**
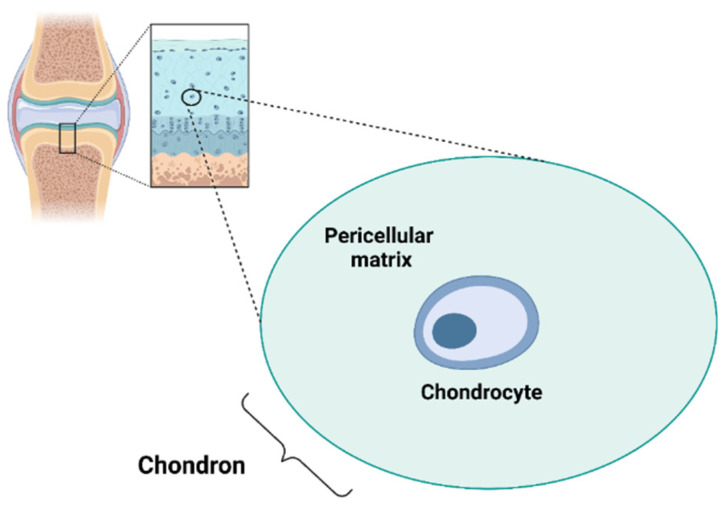
Chondrocyte and chondron. Created with BioRender.com.

Chondrocytes are the only cell type present in the AC and represent 1–5% of cartilage tissue (Figure 1).

They originate from mesenchymal stem cells (derived from embryonic cells specific to the mesenchyme) and, in adult cartilage, are quiescent, fully differentiated cells that receive nutrients via diffusion from the articular surface. They are characterised by the presence of the primary cilium, a short microtubule-rich appendage, which seems to play an important role as a mechanosensor [14,15]. In general, cell adhesion molecules, cytoskeletal elements, matrix protein receptors, integrins, the primary cilium and mechanically activated ion channels are recognised as the mechanosensory players responsible for the chondrocyte’s mechanical behaviour [16]. Regarding mechanically gated ion channels, Piezo-type mechanosensitive ion channel component 1 (Piezo 1), Piezo-type mechanosensitive ion channel component 2 (Piezo 2) and the transient receptor potential vanilloid 4 (TRPV4) are of particular interest in regulating calcium influx in chondrocytes [17]. These channels are mechanical sensors present in different cell types and they seem to be involved in different diseases [18,19,20].

Chondrocytes are responsible for the synthesis and degradation of the ECM [21]. The ECM is particularly enriched with collagen II and proteoglycans (PGs) forming networks (containing hyaluronic acid (HA), glycosaminoglycans (GAGs), chondroitin sulphate, fibres, laminin and fibronectin). Thus, chondrocytes modulate the enzymatic breakdown of the ECM maintaining a balance between anabolic and catabolic ECM processes. When the balance is disrupted in favour of ECM catabolism, progressive AC degeneration occurs that could lead to OA [22]. Chondrocyte physiology and control of matrix turnover are influenced by several environmental factors such as soluble mediators (e.g., growth factors and cytokines) and external tissue damage [23].

**Figure 2:**
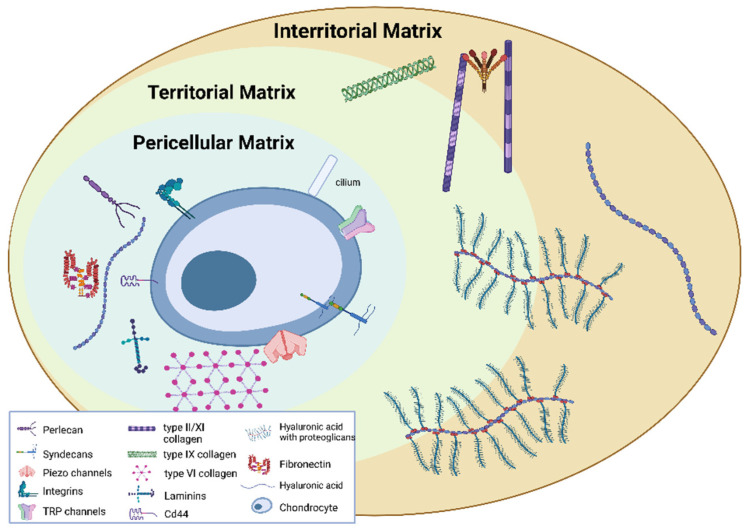
Pericellular, territorial and interterritorial matrix. Created with BioRender.com.

The ECM can be divided into the PCM, territorial matrix (TM) and interterritorial matrix (ITM), which differ in matrix composition and organisation [24]. The PCM represents a specialised thin layer of the ECM with a thickness of 2–4 µm and it is located around the chondrocyte (Figure 1). It is composed of aggrecan, HA, GAGs, and a particularly high concentration of type VI collagen, which is exclusively present in the PCM, and low or no type II collagen. The PCM plays a crucial role in the metabolic activity and mechanical properties of chondrocytes as it is involved in AC homeostasis, and in protecting chondrocytes from external stresses [25,26,27,28,29]. Indeed, the PCM acts as a protective barrier for cells, enabling them to retain the width/volume of the chondrocyte during compression and allowing the translation of the mechanical stimuli to the cells [30]. The PCM is surrounded by the TM containing type II, IX and XI collagens (Figure 2). The ITM is the largest region and it is composed of a fibrillar collagen network formed by type II collagen fibrils containing type XI collagen within the fibril and type IX collagen on the fibril surface with the non-collagen domain exposed, allowing the interaction with other matrix components. The orientation of ITM collagen fibrils is different depending on the AC zone [22,31,32]. In this regard, the AC can be divided into three zones: the superficial zone, middle zone and deep zone. The superficial zone is characterised by thin collagen fibrils running parallel to the articular surface. In the middle zone, there is no particular orientation of the collagen fibrils, while they are perpendicularly oriented to the articular surface in the deep zone [32].

## 3. Experimental Methods for the Mechanical Characterisation of the Chondrocyte and Chondron

Chondrocytes embedded in the ECM are constantly exposed to mechanical loading, and cartilage mechanobiology is modulated by their mechanical signals. For this reason, the quantification of chondrocytes’ mechanical properties can lead to a better understanding of cartilage biomechanics and mechanobiology, along with the identification of the main factors involved in their alteration [33].

The mechanical properties of chondrocytes have been quantified using several measurement methods in conjunction with theoretical models.

The most commonly used methods to evaluate single-cell mechanical properties are atomic force microscopy (AFM), micropipette aspiration (MPA), cytoindentation and micromanipulation techniques, described below.

### 3.1. Atomic Force Microscopy

The AFM is one of the most commonly applied techniques for material characterisation at the micro and nanoscale, to extract local mechanical properties of a material or to describe its microstructures and texture. The common setup is formed by a flexible cantilever beam with a tip that can have different shapes and sizes (Table 1), which should be taken into account when indenting a cell. As a matter of fact, indenting a different region of a cell or using different experimental setups (e.g., changing the tip speed or shape) can lead to significantly different results. The deflection of the cantilever beam, which represents cell deformation, is measured by a laser. AFM has become quite popular for cell mechanical testing as it combines three-dimensional imaging at the nanoscale with the nanoindentation of cells and allows measurements of various cell regions, such as its surface, its subcomponents or the whole cell [34]. When referring to chondrocytes, Darling et al. reports a mean constant displacement of about 1 ± 0.3 µm which is applied to the cell using a microscopic cantilever tip made of glass or silicon nitride, usually designed ad hoc. The force applied to the cell is determined by multiplying the cantilever stiffness (the known quantity) by its deflection. Then, from the force-displacement data, it is possible to determine the mechanical parameters of chondrocytes such as the elastic modulus as reported in Table 1.

### 3.2. Micropipette Aspiration

MPA is a versatile and widely used technique for determining the mechanical properties of living cells [39]. This is usually performed by applying a negative suction pressure by means of a pressure control system directly connected to a micropipette (diameter ranging from less than one micron to tens of microns [40]). The micropipette is positioned close to the cell surface and the negative pressure acts as an attractive force deforming the cell shape. By maintaining constant and stable pressure on the cell, it is possible to perform creep tests in which the cell relaxes inside the micropipette for a certain time. The aspiration length of the cell, inside the micropipette chamber, is recorded during the experiment until the equilibrium is reached. The micropipette technique has been used by different authors in order to investigate the elastic and viscoelastic mechanical properties of chondrocyte subcomponents ([41,42]) or to compare results obtained from different experimental methods ([35]).

### 3.3. Cytoindentation and Micromanipulation

Cytoindentation is a technique first developed for displacement-controlled indentation tests on single cells [43]. Over the years, it has been modified in order to perform creep indentation experiments on adherent cells [44,45]. This technique is widely used to perform compression tests on chondrocytes, as they are anchorage-dependent cells that usually experience compressive forces in vivo [44]. The experimental setup usually consists of a 5 µm diameter glass or tungsten flat probe that indents cells, which are attached to a glass substrate. The creep test is performed by applying a constant stress to the chondrocytes and measuring the obtained cell deformation. Different probe diameters are used, leading to a modified cytoindentation apparatus, also called the unconfined creep compression tool [45,46,47]. While in cytoindentation the tip probe is smaller than the tested cell, in the modified apparatus the flat tip has a diameter bigger than the cell (e.g., about 50 µm) [45,46]. Similarly to the latter, micromanipulation is a technique used to evaluate the mechanical behaviour of a suspended chondrocyte by compressing the single cell between two parallel surfaces, such as the flat end of a glass probe and the bottom of a glass chamber containing cells immersed in a culture medium [48,49].

## 4. Experimental Results on Chondrocytes and Chondrons

The chondrocytes considered for the experimental tests were primary cells and they were isolated from human and animal cartilage. The main method used for cell harvesting appears to be the enzymatic digestion of cartilage with pronase [41], collagenases [34,38,44,45,46,50] or with both enzymes [35,36,39,41,42,49,51,52,53,54]. More details are reported in Section 5.1. The incubation time of digestion ranged between 1 and 16 h at 37 °C.

Researchers of the majority of the analysed studies performed the tests at room temperature keeping cells immersed in phosphate-buffered saline (PBS) in order to prevent sample dehydration.

Regarding the chondron [55], the role of PCM components (i.e., collagens, PGs and GAGs) in the mechanical response is still unclear. Wilusz et al. [56] used different enzymes to digest specific GAGs and PGs (i.e., aggrecan, dermatan sulphate/chondroitin sulphate and hyaluronan) in cryosections of porcine cartilage in order to investigate their impact on the biomechanical behaviour of the ECM and PCM. They observed that, regardless of the digestive technique, only the ECM’s elastic moduli were reduced. Elastase has been shown to degrade both PCM and ECM, and thus could cause a decrease in their elastic moduli. However, the authors demonstrated that PCM was not as affected as ECM was thanks to its resistance to other enzymes during digestion.

### 4.1. Influence of the Site and Depth

An important factor to be considered in the mechanical properties of chondrocytes and chondrons is the cartilage zone in which cells are embedded.

Wilusz et al. [27] tested the region between 0.2 and 0.4 mm from the articular surface (which corresponds to the middle–upper deep zone) through AFM indentation. They produced 5 mm thick slices of cartilage samples from femoral condyles, sectioned perpendicular to the articular surface, by adopting a cryostat microtome, in order to evaluate the mechanical properties of the ECM and PCM in situ at different depths. Moreover, with reference to chondrocytes, many studies were conducted testing cells harvested from the surface, middle and deep zone of cartilage [35,46,57]. Indeed, these authors agreed that superficial cells have significantly higher moduli and apparent viscosity than middle/deep ones do. This variation was supposed to be influenced by different loading histories experienced by cells in each zone [46]. Moreover, it was shown that cells differ not only in mechanical behaviour, but also in size, volume and shape depending on the zone. A summary of chondrocytes’ mechanical parameters obtained within the analysed studies is reported in Table 2.

Differently from the chondrocyte, the chondron does not exhibit significant differences between sites and depths in terms of both mechanical response and mechanical properties [58,59,60]. On the contrary, the morphology and orientation of the chondron may change significantly depending on depth, as reported by Youn et al. [61] who investigated the chondron structure along the whole thickness of the AC (from the surface to the deep cartilage) of a porcine knee. Chondrons located at the superficial layer presented a discoidal flattened shape oriented parallelly to the surface of the cartilage, while chondrons located at an intermediate level were more rounded and did not exhibit a preferred orientation. Lastly, chondrons located at a deeper level revealed groups in which a single PCM was able to encapsulate multiple chondrocytes creating an oval-shaped structure oriented perpendicularly to the cartilage surface [61].

### 4.2. Human vs. Animal

Chondrocytes were isolated from cartilage harvested from different sites, such as knees and hips [36,39,51,53,54], for human cells (donors aged between 20–86 years), while animal cells were harvested from femoral condyles and distal metatarsal joints from different sources, such as rat [62], dog [57], pig [35,41] and cattle, which included cows, calves and steers [34,38,42,44,45,46,47,48,49,50,52,63,64,65].

Comparing the elastic modulus, *E*, of human and animal chondrocytes, it was observed that human chondrocytes range between 0.65 and 1.4 kPa [36,39], while greater variability was observed among different species (*E* ranging between 0.97 and 23.9 kPa [34,35,38,42,44,45], as reported in Table 2), but always within the same order of magnitude. Moreover, both human and animal chondrocytes were obtained from various joints; this aspect represents another key variable in the evaluation of biomechanical properties as it is likely that chondrocytes of different joints might have different biomechanical properties.

As observed in Table 3, the mechanical properties of chondrons from different animal species are significantly different.

Darling et al. [59] demonstrated that the ratios between the mechanical properties (namely their Young’s Moduli) of the PCM and the ECM are constant and in the range of 0.34–0.37 for all species included in the study (human, rats and pigs). However, no details were provided regarding this ratio. It is unclear if it was obtained by averaging the mechanical properties of the ECM at different depths or if it was limited to AFM indentation tests.

## 5. Factors That May Influence the Experimental Results

The first factor to be considered is the different testing configurations, such as AFM, cytoindentation, micromanipulation and MPA.

AFM and cytoindentation techniques are performed on chondrocytes attached to a substrate; in the former case, cells are usually seeded on poly-L-lysine coated slides [35,37], while in the latter, chondrocytes are usually attached to a glass slide [44,46,47].

MPA and micromanipulation procedures are used to assess the mechanical properties of cells in suspension [35,48,49], even if MPA could allow a test of both suspended and adherent cells. The elastic and viscoelastic properties could be obtained on suspended cells [35,39,51,53,54], while the adhesion force can be investigated at the single-cell level on adherent cells [69].

Experimental tests can lead to different mechanical parameters depending on the adoption of suspended or adherent cells. Some studies compared AFM and MPA tests on chondrocytes and stated that no differences were found [35]. On the contrary, other studies asserted that some differences were found if cells were tested in suspension or attached to a substrate. Indeed, micromanipulation [48,49], performed on suspended cells, and modified cytoindentation tests [45,46], performed on adherent chondrocytes, lead to different mechanical parameters, even if both procedures compress the entire cell.

Further differences emerged comparing several literature studies: MPA [51], AFM [35] and cytoindentation [44] deform only a portion of the cell’s membrane, while micromanipulation [48,49] and modified cytoindentation [45] compress the whole cell into a large nominal strain. Moreover, MPA and AFM usually give information about local mechanical properties, e.g., the cell membrane, while micromanipulation reflects the mechanical properties of whole cells and of their subcomponents, such as the cytoskeleton and nucleus [49].

As previously reported, observing Table 2, the parameters of chondrocytes are different but in the same order of magnitude. This could be attributed to different testing methods but also to the inhomogeneity of the cell structure; indeed, the nucleus contributes considerably to unconfined compression, while it plays a smaller role in the tensile response to MPA [45].

Differences induced by the test setup have been also been found considering two studies, which adopted cells harvested from the same species and joint; cytoindentation [44] and modified cytoindentation [46] were performed with the same device, but with slightly different procedures since the first one is an indentation procedure, while the second one is an unconfined compression. Comparing the results obtained via these two testing methods, it was observed that the instantaneous Young’s modulus obtained with the cytoindentation technique was about eight times higher than that obtained with the modified cytoindentation one.

Chondrocytes’ mechanical properties can also be influenced within the same technique, by varying the setup. In AFM experiments, different probe sizes (micrometers or nanometers) and tip shapes, e.g., spherical or pyramidal [34,35,36,37] are available. As shown in Table 1, by varying the tip radius, authors found consistent changes in the Young’s modulus; more precisely, with a sharp nanosized tip the elastic modulus resulted to be higher than that obtained with a larger spherical probe [70,71]. This could be due to the fact that, using a sharp tip to indent a cell, the tip first encounters different cytoskeleton rearrangements under the cell membrane [34], and second, the chondrocyte volume indented and investigated via the AFM tip is different [38]. Moreover, the hydraulic permeability, *k*, is slightly different between experiments using a nanosized and those using a microsized tip probe [37,38].

### 5.1. Sample Harvesting Techniques and Culturing

Regarding chondrocytes, the harvesting technique is not considered a factor that can influence their mechanical properties, even if it could have an impact on the cell biomechanical parameters due to possible cell damage. Moreover, the harvesting method adopted in all the testing procedures reported in the studies analysed in this review is only the enzymatic one [34,35,36,38,39,41,42,44,45,46,49,50,51,52,53,54]. After the harvesting procedure, chondrocytes are cultured in different ways depending on the testing method adopted to assess their mechanical properties. They are cultivated in alginate beads if cells are tested with the MPA technique [39,41,51,53] or attached to a substrate if they are tested with the AFM [34,36,37,38] or cytoindentation procedure [44]. In the first case, chondrocytes are suspended in the beads in culture media (DMEM [39] or Ham’s F-12 medium [41] with FBS and penicillin/streptomycin) until tested. Immediately prior to testing, the alginate beads are dissolved in sodium chloride and sodium citrate to release the chondrocytes which are then suspended in Hank’s balanced salt solution [39,51,53] or in Dulbecco’s phosphate-buffered solution [41], containing bovine serum albumin and sodium chloride/sodium citrate solution [39,41,51,53]. In the second case, cells are suspended in culture media (composed of DMEM, penicillin, streptomycin and FBS) and then seeded on poly-L-lysine-coated polystyrene plates [34,35,37,38]. No studies investigated the influence of different culture methods on biomechanical parameters as well as different testing methods. Therefore, specific studies comparing different culturing and storage methods are needed in order to better clarify this point and to identify the best culture system for mechanotransduction tests.

Chondrons showed significantly different mechanical responses depending on the harvesting technique used. Two techniques are typically adopted to isolate chondrons: enzymatic digestion of the territorial matrix (using dispase and collagenase) and a mechanical homogenisation process. The former leads to relatively abundant yields, while the mechanical extraction process reduces the number of viable chondrons [72].

Mechanical tests of isolated chondrocytes (ICs), mechanically extracted chondrons (MCs) and enzymatically extracted chondrons (ECs), all from the middle/deep layers of canine AC, led to significantly different mechanical responses [72]). The MCs resulted in a stiffer global response compared to that of the ICs and ECs. Indeed, MCs resulted in being stiffer compared to the ICs and ECs embedded in agarose gel via compression experiments [72]. This difference appeared to become less evident over time, as incubating the samples for up to 7 days led to the partial reconstruction of the PCM around the ICs and MCs and thus to a more similar response between the three groups. This observation supports the hypothesis that the enzymatical extraction process leads to a degraded/damaged PCM in ECs. This point was further confirmed via osmotic challenge tests performed on ICs, ECs, and MCs [28]. When exposed to a hypertonic solution, the ICs and ECs shrank more than the MCs did and the difference in swelling was reduced by culturing the samples for up to one week. These results together with the concept that the PCM is believed to be involved in protecting the chondrocyte from osmotic changes support that enzymatic isolation tends to isolate chondrons with a damaged PCM compared to the MCs.

Therefore, the ECs exhibit a behaviour resembling that of ICs, which completely lack the PCM structure [28,72], and this is supported by several published studies aimed at comparing the physiological or mechanical behaviour of ICs and ECs [49,73].

Chondrons are usually cultured using different methods: alginate beads [67], agarose gel [73] or assembly in pellets [74]. It should be noted that culturing chondrons tends to override the impact of the isolation method used (mechanical vs. enzymatic) as the chondrons restore a new complete PCM in vitro when the proper nutrients and growth factors are added to the culture media. Although the differences between ECs and MCs tend to disappear after 3 weeks in culture [74], the cultured chondrons do not stop the development of a functional and complete PCM even after 28 days of culture [67]. Thus, the ability of a chondrocyte to build a proper PCM could be exploited to solve some of the problems related to the extraction method used. It is worth mentioning that it is currently unknown how long it takes for ECs and ICs to form a fully functional PCM in vitro, since studies [67,74] pointed out the achievement of a final PCM stiffness lower than the one of freshly mechanically extracted chondrons, thus raising doubts about this possibility.

In conclusion, a less functional PCM in ECs makes them behave more similarly to ICs in mechanical tests. Conversely, MCs tend to better preserve the PCM, thus leading to a stiffer chondron which behaves differently from ECs and ICs.

### 5.2. Influence of the Mechanical Test

Two main mechanical tests were performed on chondrons: AFM and MPA. In Table 3, the parameters used to describe chondron behaviour in the literature are reported. Both animal and human tests were included due to the paucity of published data.

The AFM indentation tests may provide information about the chondron when it is still embedded in the surrounding ECM using a stiffness mapping method. On the other hand, this approach lacks lateral resolution and thus, the obtained measures could be affected by the presence of the TM and ECM [27,66,67]. A possible solution was suggested in the literature such as the use of a conservative approach when identifying the PCM which reduced the sample area for parameter estimation [59]. Another problem with the AFM is the choice of the right indenter size to use; a nanometric indenter can be more precise in terms of area investigation but tends to cause the artificial stiffening of the sample if compared to micrometric indenters [59].

AFM indentation can also be used to test cells adherent to a substrate as well as those captured in microwells [67]. They tested single chondrocytes isolated from calves and cultured in a monolayer. Moreover, they compared the results obtained from isolated chondrocytes with 3D chondrocyte cultures (chondrocytes cultured in 3D alginate gels for 28 days to favour the production of their own PCM). Although the authors stated that 28 days was not sufficient to fully develop a complete PCM, the plateauing of the mechanical response was considered a good indicator of a mechanically functional PCM [67]. Wilusz et al. [27] observed different mechanical properties for chondrons located on the lateral and medial human condyles, where the PCM of chondrons located in the lateral condyle appeared to have a stiffness value of 30% less than that of those located in the medial condyle.

The MPA technique applies a tensile stress to the surface of the sample and usually requires cells to be suspended in a medium. This method can be used to test viscoelastic properties as well as the elastic response of the specimen.

The studies of Alexopoulos [58] and Guilak [60] showed that the use of a half-space model might lead to an underestimation of the mechanical parameters when compared to the use of a layered or shell model due to the fact that the half-space model does not take the geometry and compressibility of the chondron into account.

Finally, it is worth mentioning that although these authors stated that the experimental data revealed a possibly viscoelastic response, only a few studies tried to characterise this type of response with a biphasic [68] or viscoelastic [49] model.

### 5.3. Sample Storage

Although several studies did not report the storage method used, when reported, different storage strategies were observed for chondrons depending on the type of experiment performed. When dealing with AFM stiffness mapping, the cartilage samples were frozen at −20 °C, wrapped in phosphate-buffered saline (PBS) gauzes and then the cartilage samples were cut to obtain slices for AFM stiffness mapping [27,56,59,66]. Other less common procedures were the use of fresh sample slices [25], and fixation with 4% paraformaldehyde followed by decalcification in 10% EDTA for 21 days [75]. When performing MPA tests, the chondrons were extracted from the cartilage and usually stored in a glass container (such as a petri dish), covered with PBS for the immediate tests [60].

## 6. Theoretical Models for Cells Biomechanics

Chondrocytes’ mechanical properties may differ also depending on the adopted model used to describe cell behaviour and to fit experimental data obtained from a specific experiment.

The common approaches used to describe chondrocytes’ material behaviour are the elastic or the viscoelastic one, as reported in many studies (e.g., [26,35,44] to cite a few).Viscoelasticity is also able to capture the viscoelastic effects that usually emerge during the creep and stress–relaxation behaviours of a cell.

When a cell is compressed until a large deformation occurs, non-linear elasticity appears to be more suitable, thanks to the adoption of hyperelastic laws to take this aspect into account [37,38,49], as is carried out with the neo-Hookean material model.

A biphasic formulation was also introduced to consider the contribution of two physical mechanisms: the intracellular fluid flow inside the cell, representing the cytoplasm, and the solid components, such as cytoskeleton and organelles [38,45]. Some studies used models of increasing complexity, by combining two or more of the previous material models, in order to better describe the real behaviour of cells. These models are the viscohyperelastic, porohyperelastic and the poroviscohyperelastic ones [38]. However, it has been reported that similar values have been obtained for the cell modulus from the elastic, viscoelastic and biphasic models [35,36,44,45,46]. As is possible to see from Table 2 and from the literature [38], the Young’s modulus (which describes the elastic behaviour of chondrocytes) obtained with a model of increasing complexity falls in the same range (0.62–27 KPa) of those obtained using simpler mechanical characterisation techniques [26,35,39,44,45,46,47,51]. However, with a model of increasing complexity, such as the viscoelastic, hyperelastic or biphasic models, it is possible to describe not only the linear elastic behaviour of the cell, but also the non-linear (hyperelastic), time-dependent response (viscoelastic) and the contribution of the fluid and solid phase inside the chondrocytes itself.

In many material models, chondrocyte behaviour is assumed to be incompressible (Poisson’s ratio, *ν* = 0.5 [36,41,44,45,49]), as reported in Table 4, even if it has been demonstrated that cell incompressibility is not valid under direct compression, e.g., that in modified cytoindentation tests [46]. Otherwise, the use of the compressible hypothesis leads to different results, such as a higher relaxed modulus and apparent viscosity [34,38,46].

Several theoretical models have been used to fit the experimental data in order to determine chondrocytes’ properties. Force–displacement data obtained with the AFM technique are usually fitted with the Hertz contact model [34,35,50] or with a thin-layer Hertz model [36,76]. The first one describes the interaction between two spheres, the cell and the cantilever tip; the second one represents a hard sphere (the cantilever tip) indenting a flat deformable substrate (the cell). Experimental data gained with MPA tests are usually fitted with Theret’s model [77], to obtain the elastic cell parameter [39,42,52], or with the theoretical model formulated by Sato [78], which considers time dependence [35,51,53,79]. In addition, the standard linear solid model was employed to determine the viscoelastic properties of chondrocytes during relaxation tests, performed with AFM [35,36] or micromanipulation [49] and during creep tests, conducted with MPA [35,51,79] or cytoindentation [44,45,46]. All these analytical models have the advantage of being easily applicable. Indeed, they rely on the linearity of the chondrocytes’ mechanical responses as they consider cells formed only by a single solid component. To evaluate the parameters of a cell which shows viscohyperelastic behaviour, a model has been developed named the standard neo-Hookean solid model (SnHS), proposed by Zhou [80]. This model was later modified (mSnHS) in other to capture the strain rate-dependent mechanical behaviour of both living and fixed cells [37].

## 7. Mechanical Role of the Subcomponents

### 7.1. Mechanical Role of the Cellular Subcomponents

Usually, cells are modelled as solid homogeneous materials in order to simplify their behaviour, thus losing the role of different subcomponents such as the nucleus, cytoskeleton and organelles. For this reason, some studies investigated this aspect, which highlighted important insights such as the fundamental structural role of the cytoskeleton in cells’ behaviour [51,53]. By comparing cells after a few hours (usually 2 h) and 2–3 days in culture, it was shown that the first ones were stiffer than the others. This can be due to the structural alterations of the cell’s cytoskeleton, which occur leaving the cells in culture for a longer period [34,37,76]. Since the cytoskeleton is composed of microfilaments, intermediate filaments and microtubules, several studies also investigated their contribution, by adding a specific disruptive agent for each cytoskeleton component, before the mechanical test. Different testing methods have been adopted, e.g., MPA, modified cytoindentation and micromanipulation [42,47]. Thanks to the unconfined compression testing method, achieved via modified cytoindentation, it was observed that each cytoskeletal component contributes differentially to the compressive properties of single chondrocytes. More precisely, actin microfilaments contribute to bulk cell compressive stiffness. Indeed, it was demonstrated that its disruption led to a decrease in the cell’s compressive modulus (from 1.63 ± 0.31 kPa for a cell with a functional cytoskeleton to 1.01 ± 0.10 kPa for a cell without actin microfilaments). Instead, intermediate filaments play an important role in cellular compressibility and microtubules contribute to the incompressible nature of cells. Due to the disruption of the microtubules inside the cell, the Poisson’s ratio changed, from a value of 0.49 for an intact cell to 0.36 for a chondrocyte without the microtubules [47].

Williantarra et al. showed that the substrate stiffness affected centriole positioning, cell morphology, actin architecture and primary cilium length in murine chondrocytes [81].

Importantly, it has been shown that the depletion of primary cilia in murine tibial cartilage impacts the mechanical stiffness. In particular, the cartilage of these mice had lower instantaneous and equilibrium moduli (approximately half of those observed for wild-type cartilage) [82].

Moreover, the mechanical properties of the chondrocyte nucleus have been investigated. Highly significant differences were found between the properties of single chondrocytes and those of isolated nuclei [41]. MPA tests showed that mechanical properties of chondrocytes’ nuclei are different from those of the cytoplasm and they are stiffer and more viscous than are intact cells.

Regarding calcium channels, TRPV4-mediated Ca^2+^ signalling played a central role in the response of chondrocytes to low (physiological) levels of strain (3% and 8% of strain), while Piezo channels played a central role in the response of chondrocytes to high strain (traumatic) levels (18% of strain) [83].

### 7.2. Mechanical Role of the Major ECM Subcomponents

As described earlier, the ECM and PCM are composed of different molecules, which contribute to their biochemical and mechanical properties. In the ECM, the main component is type II collagen, which makes up to 90–95% of the cartilage collagen. This type of collagen is mainly produced by the chondrocyte and is organised in complex scaffolds able to sustain the mechanical forces that the AC is usually subjected to. Two other important components of the ECM of the AC are type XI and type IX collagen. The former represents 3% of adult AC, while it forms up to 10% of foetal cartilage. This type of collagen is the first one to be synthesised by stem cells differentiating into a chondrocyte and it is usually found close to this cells’ surfaces. This arrangement leads to the belief that type XI collagen plays a role as a mediator between the PCM and chondrocyte by interacting with PGs present in the cartilage [84]. Type IX collagen on the other hand contributes to only the 1–5% of the total collagen in adults and it is believed to stabilise the organisation of fibrils and proteoglycans thanks to its lateral association with both collagen type XI and type II.

Furthermore, a reduction in the quantities of type IX collagen present in the cartilage is linked to different pathological states. It is believed that a reduction in the amount of this type of collagen, for example due to ageing, could contribute to the development of osteoarthritis [84].

Another important type of collagen present in articular cartilage is type VI collagen. This type of collagen is present in most tissues of the human body but in articular cartilage it can only be located in the PCM. The ability of this type of collagen to interact with many of the constituents of the ECM hints at its role in anchoring the chondrocyte to the PCM and the spatial organisation of the ECM relative to that of the PCM [84]. Some studies have highlighted how a deficiency of type VI collagen leads to an accelerated development of OA, thus further reinforcing the link between the organisation of its network to the mechanotransduction processes of chondrocytes.

## 8. Pathological Changes in Osteoarthritis

Osteoarthritis (OA) is the most common form of arthritis; it is considered a leading cause of disability among older adults and a major public health concern [85]. The knee is the joint most frequently affected by OA followed by the hand and hip [86,87].

Historically, OA was considered a disease involving only the AC. During the last few years, the concept of OA has changed to one of it as a multifactorial whole joint disease involving not only cartilage but also meniscal degeneration, subchondral bone remodelling, inflammation and fibrosis of both the infrapatellar fat pad and synovial membrane [6,88,89,90,91,92,93,94,95,96]. OA changes determine the alteration of the biomechanical behaviour of different joint tissues [6,91,96]. Among the articular tissues, the AC plays a fundamental role in withstanding mechanical stress as it provides load-bearing surfaces along with low friction and wear resistance, and gliding properties [6,97,98]. Furthermore, the AC allows the support and redistribution of the compressive, tensile and shear forces originating during joint articulation [99,100,101].

Several risk factors are associated to OA including age, sex, obesity, ethnicity, genetics and previous history of injury or joint trauma such as meniscal damage [100,101,102]. Among the different risk factors associated with OA development, ageing plays a significant role and it should be noted that joint ageing and OA are not the same but ageing changes can facilitate the development of OA [103]. During ageing, the AC becomes thinner with a slightly brown appearance due to advanced glycation end-products that modify the biomechanical behaviour of the tissue [103]. OA development and progression are also supported by chronic low-grade local and systemic inflammation through the release of inflammatory molecules affecting chondrocytes’ structural and metabolic activities [104]. Due to OA onset and development, the AC undergoes structural remodelling driven by many factors including mechanical stresses (wear and tear due to an increase in the superficial roughness [105]), genetic predisposition and low-grade inflammation [89,106,107,108]. The main cellular events underlying OA cartilage destruction are ECM fibrillation and degradation secondary to mechanical breakdown and the up-regulation of matrix-degrading enzymes triggering a proinflammatory cascade, collagen denaturation (especially type II collagen) and the loss of PGs resulting in a softer ECM [108,109,110]. Moreover, inflammatory cytokines and other molecules released by the synovial membrane stimulated by damage-associated molecular patterns determine the instauration of a vicious cycle, which leads to cartilage degeneration [111].

Chondrocytes acquire a hypertrophy-like phenotype determining an altered matrix production coupled with an increase of matrix-degrading enzymes’ expression (i.e., metalloproteinases [112]). In early OA, there is an attempt to regenerate/repair the matrix by increasing the synthetic activity (increased ratio of collagen/aggrecan [113,114]). However, it induces PG leakage and type II collagen degradation in the cartilage superficial zone with an increase in water content determining a reduction in ECM tensile strength [115]. As OA progresses, chondrocytes appear to be organised in clusters. At late stages of OA, there is a decrease in chondrocyte density because of chondrocytes’ death. Interestingly, only few cells show evidence of classical apoptosis, while the majority undergo apoptosis in a non-classical manner (expansion of the rough endoplasmic reticulum and Golgi apparatus, frequent autophagic vacuoles, extrusion of cellular material into the extracellular space and final disintegration of cell remnants) called “chondroptosis” [116,117]. An increased number of chondrocytes display a senescence-associated phenotype [118]. The primary cilia change the orientation only in superficial cartilage resulting in them being oriented away from the articular surface in normal healthy AC and to the centre of abnormal cell clusters in OA cartilage [119]. Moreover, there is an increase in cilia length and the overall percentage of ciliated chondrocytes supporting impaired signalling in OA cartilage [120].

It has been shown that Piezo1/TRPV4 communicate with each other (Piezo1 activation inhibits subsequent TRPV4 activation to a greater extent than is the case vice versa), which seems to be altered in OA chondrocytes altering metalloproteinases, collagen and interleukin gene expression [121].

An OA PCM structure appears disrupted with an altered composition determining changes in the mechanical function also [122]. Interestingly, chondrocyte proliferation and cluster formation seem to be preceded by early changes in the collagen and proteoglycan distribution of the PCM in the chondron that appears to be enlarged and loosely organised [123,124]. In particular, there is an up-regulation of type VI collagen showing zone-dependent expression [124].

## 9. Correlations between OA and Changes in the Mechanical Properties

### 9.1. Changes at the Cellular Scale

Over the years, both non-OA and OA chondrocytes’ mechanical properties were investigated, leading to contrasting results, as is possible to appreciate from data reported in the literature. Some studies reported that chondrocytes’ properties are nearly identical for cells isolated from healthy and OA cartilage [39,54]. In 2000, Trickey et al. [51] showed that OA cells were stiffer (the elastic modulus of OA chondrocytes is 1.5 times higher than that of healthy ones) and more viscous than the healthy ones (OA cells have a viscosity, which is about double the value of that reported for healthy cells), while in 2004, they [53] reported that OA chondrocytes seemed to have a lower elastic modulus and viscosity than the healthy ones did. Actually, it is still not established if these contrasting and different results could be related to pathology or to the different experimental conditions (e.g., sample storage, different isolation methods and/or conditions of culture).

Hsieh et al. supported the evidence that OA chondrocytes are less stiff than are healthy ones. They determined cell stiffness through AFM indentation and found that OA chondrocyte stiffness was 0.0347 ± 0.0005 N/m, while that of healthy ones was 0.09620 ± 0.009 (which was about three times higher with respect to the OA ones) [125].

Interestingly, porcine chondrocytes stimulated with IL1-alpha display an increase in p*PIEZO1* expression, which causes an increase in Ca^2+^ levels in the cells and attenuates the dynamics of the F-actin cytoskeleton (decreasing the mechanical stiffness of the cell), leading to an increase in the mechanosensitivity of chondrocytes to mechanical loading [126,127]. More precisely, the decrease in chondrocyte’s stiffness resulted in increased cellular deformation in response to mechanical loading [126]. Interestingly, Young et al. investigated the role of Piezo channels in OA mice demonstrating that the deletion of both genes does not impact normal joint development and has limited effects on OA progression [128].

### 9.2. Changes at the Chondron Scale

Chondrons’ mechanical properties were demonstrated to be affected by OA. As can be observed from Table 5, the Young’s modulus of an OA chondron is significantly lower than that of a healthy one in all tests performed. In general, OA is responsible for a loss of the elastic modulus of the PCM of about 30–50% in an OA AC [27,58,68].

In the early stages of OA, the importance of the mechanical properties at the chondron level is further reinforced by a finite element study by Khoshgoftar et al. [129]. They observed that the strain fields can be changed significantly around the chondrocyte within the chondron by changing only the mechanical parameters of the PCM and keeping the remaining tissue unaltered.

As explained earlier, Wilusz et al. [27] observed that some differences can be observed between chondrons located in the lateral and medial condyle of about 30%. In the same study, the authors showed that the onset of OA causes a reduction in the stiffness of chondrons’ PCM located in the medial condyle which is not present for the PCM of chondrons in the lateral condyle. According to their findings, the loss in mechanical properties makes the distinction between the PCM of medial and lateral condyle not statistically significant [27]. Alexopoulos et al. used different models (elastic layered half-space model and linear biphasic model) to characterise the material behaviour of chondrons [130]. Similarly, from what was seen for healthy chondrons, the authors stated that the half-space model underestimates the value of the elastic modulus [58], while they claimed that the biphasic model is an overall better representation of chondron behaviour as the latter takes into account the compressibility and finite geometry of the chondron. Furthermore, this second approach shows an increase in the chondrons’ permeability together with the expected decrease in stiffness [68]. Precious insights could be collected also from computational models reported in the literature. Most of the studies reinforced the hypothesis that the PCM is a fundamental structure in mechanosensing and thus exerts a key role in the mechanotransduction of external stimuli [26,129,130]. Alexopoulos et al. and Khoshgoftar et al. pointed out an increase in the local deformation of the cell due to the mechanical changes in the OA PCM, while Guilak et al. stated that it was reduced in favour of increased applied stress [26,129,130]. Julkunen et al. tried to develop a complex model of cartilage tissue using a hierarchical approach accounting for both the macroscopic and microscopic structure as well as the differences that can be found at different depths [131]. The model was not able to reproduce the same results obtained from experimental analysis but pointed out the lack of a full description of the material and methods reported in experimental studies [131].

## 10. Critical Points, Future Perspectives and Conclusions

Chondrocytes/chondrons are embedded in the ECM/PCM and constantly exposed to mechanical stimuli. The mechanical properties of these cells have been quantified using several measurement methods in conjunction with theoretical models as described in this review. The most commonly used methods to characterise their biomechanical behaviour are AFM, MPA, cytoindentation and micromanipulation techniques.

Interestingly, chondrocytes exhibit different morphologies as well as different mechanical properties depending on the different cartilage zone. Superficial cells retain a significant higher moduli and apparent viscosity compared to middle/deep chondrocytes. On the contrary, no differences were highlighted between the cartilage site and depth in terms of mechanical response and properties regarding chondrons.

The elastic modulus, *E*, of human chondrocytes ranges between 0.65 and 1.4 kPa, while greater variability is present when evaluating chondrocytes isolated from different animals (*E* ranging between 0.97 and 23.9 kPa). Indeed, it has been reported that the ratio of the PCM to ECM stiffness of the chondrons remains constant among the species and usually in the range of 0.34–0.37, even if the mechanical parameters could be influenced by the different techniques as well as the different setups used.

The cytoskeleton, the cilium and calcium channels are the main subcellular components involved in the biomechanical response of the cells.

In pathological conditions, i.e., OA, chondrocytes and chondrons are subjected to several changes that also modify biomechanical behaviour. OA chondrocytes seem to have a lower elastic modulus and viscosity compared to healthy chondrocytes. OA chondrons acquire a lower Young’s modulus compared to healthy chondrons.

Moreover, most of the studies reported only partial information regarding the depth, origin, methods used for cell/chondron isolation, conservation and histological analysis of the samples. The lack of this information limits not only the reproducibility but also the comparison of data among different studies. Moreover, it hinders the development of computational models. This is exemplified by the study of Nguyen et al. [49]. They were able to compellingly reproduce experimental findings using a FE model supporting the importance of having access to the complete experimental information and variables.

In conclusion, this review not only summarises the description of chondrocyte and chondron mechanical properties, but also underlines the strong influence of all the other direct and indirect variables, which play a key role in planning an experimental protocol as well as in comparing the results of different studies. The quantification of cells’ mechanical properties can lead to a better understanding of cartilage biomechanics and mechanobiology, along with the identification of the main factors involved in their alteration.

## Figures and Tables

**Table 1:** Effect of elastic modulus of chondrocytes on varying tip radius in AFM tests.

Reference	Tip Radius	Tip Shape	Elastic Modulus (kPa)
[35]	2.5 µm	Spherical	*E_elastic_* = 1.27 ± 0.61
[36]	2.5 µm	Spherical	*E_elastic_* = 1.4 ± 1.1
[34]	20 nm	-	*E_elastic_* = 10 ± 4.1
[37]	2.5 µm	Colloidal	*C*_1_ = 0.669 ± 0.365
[38]	35 nm	Spherical	*E* = 10.9 − 23.9

Elastic modulus is reported as mean ± standard deviation.

**Table 2:** Chondrocyte mechanical parameters obtained using different testing methods and material models.

Mechanical Test	Origin	Reference	Cell Source	Cartilage Depth	Material Model	H/OA	Parameters
AFM	Human	[36]	Femoral heads, ages: 34–47 years	full thickness	Linear elastic	H	*E* = 1.4 ± 1.1
					Viscoelastic		*E*_0_ = 0.914 ± 0.967
							*E*_∞_ = 0.45 ± 0.44
							*µ* = 4.5 ± 3.74
		[37]	N/A	N/A	Viscohyperelastic	H	*C*_1_ = 0.669 ± 0.365
							*D*_1_ = 4.06 ± 2.4 (×10^−3^)
							*g*_1_ = 0.6 ± 0.14
							*k*_1_ = 99.76 ± 0.08 (×10^−2^)
							*τ*_1_ = 0.082 ± 0.002
	Animal	[35]	femoral condyles; 2–3 year--old pig	Superficial	Linear elastic	H	*E* = 1.27 ± 0.61
					Viscoelastic		*E*_0_ = 0.55 ± 0.23
							*E*_∞_ = 0.31 ± 0.15
							*µ* = 1.15 ± 0.66
				Middle/ deep	Linear elastic		*E =* 0.61 ± 0.34
					viscoelastic		*E*_0_ = 0.29 ± 0.14
							*E*_∞_ = 0.17 ± 0.09
							*µ* = 0.61 ± 0.69
		[34]	femoral condyles; 13–22-month-old bovine	full thickness	Linear elastic	H	*E* = 10 ± 4.1
		[38]	femoral condyles; 18–22-month-old bovine	full thickness	Porohyperleastic	H	*E* = 23.9
							*k* = 0.08 × 10^−16^
					Viscohyperelastic		*E* = 11.9
							*g*_1_ = 0.55
							*τ*_1_ = 6
					Poroviscohyperelastic		*E* = 10.9
							*k* = 0.66 × 10^−16^
							*g*_1_ = 0.55
							*τ*_1_ = 15
MPA	Human	[39]	knees, hip, ankles and elbows; ages: 37–83 years old	full thickness	Linear elastic	H	*E* = 0.65 ± 0.63
						OA	*E* = 0.67 ± 0.86
		[51]	knees and hips, ages; 28–86 years old	full thickness	Viscoelastic	H	*E*_0_ = 0.41 ± 0.17
							*E*_∞_ = 0.24 ± 0.11
							*µ* = 3.0 ± 1.80
						OA	*E*_0_ = 0.63 ± 0.51
							*E*_∞_ = 0.33 ± 0.23
							*µ* = 5.8 ± 6.5
		[53]	femoral heads, ages; 20–79 years old	N/A	Viscoelastic	H	*E*_0_ = from 0.45 ± 0.2 to 0.7 ± 0.6
							*E*_∞_ = from 0.2 ± 0.1 to 0.3 ± 0.23
							*µ* = from 7.8 ± 8 to 9.5 ± 10
						OA	*E*_0_ = from 0.52 ± 0.25 to 0.65 ± 0.4
							*E*_∞_ = from 0.25 ± 0.13 to 0.28 ± 0.18
							*µ* = from 4.8 ± 5 to 10.1 ± 15
	Animal	[41]	femoral condyles; 2-year-old pig	N/A	Viscoelastic	H	*E*_0_ = 0.43 ± 0.07
							*E*_∞_ = 0.18 ± 0.05
							*µ* = 2.5 ± 1.80
		[35]	femoral condyles; 2–3-year-old pig	Middle/ deep	Viscoelastic	H	*E*_0_ = 0.45 ± 0.14
							*E*_∞_ = 0.14 ± 0.05
							*µ* = 2.57 ± 1.83
		[42]	metacarpal phalangeal joints	full thickness	Linear Elastic	H	*E* = 0.97 ± 0.45
Cytoindentation	Animal	[44]	distal portion of the first metatarsal; cow	full thickness	Linear elastic	H	*E* = 1.10 ± 0.48
					Viscoelastic		*E*_0_ = 8.0 ± 4.41
							*E*_∞_ = 1.09 ± 0.54
							*µ* = 1.50 ± 0.92
Modified Cytoindentation	Animal	[45]	distal metatarsal joint; 1–2-year-old heifers	Middle/ deep	Linear elastic	H	*E* = 2.55 ± 0.85
					Viscoelastic		*E*_0_ = 2.47 ± 0.85
							*E*_∞_ = 1.48 ± 0.35
							*µ* = 1.92 ± 1.80
					Biphasic		*H_A_* = 2.58 ± 0.87
							*k* = 2.57 × 10^−12^
		[46]	distal metatarsal of 12–24-month-old heifers and steers	Superficial	Viscoelastic	H	*E*_0_ = 1.20 ± 1.00
							*E*_∞_ = 0.80 ± 0.55
							*µ* = 3.75 ± 9.46
				Middle/ deep			*E*_0_ = 0.78 ± 0.38
							*E*_∞_ = 0.64 ± 0.31
							*µ* = 3.18 ± 7.33
Micromanipulation	Animal	[49]	trochleal humerus; 18-mont-old cows	Full thickness	Non-linear elastic (hyperelastic)	H	*E* = 14± 1.0
					Non-linear viscoelastic (viscohyperelastic)		*E*_0_ = 21 ± 3
							*E*_∞_ = 9.3 ± 0.8
							*µ* = 2.8 ± 0.5

For each analysed study, the following information is reported: origin which can be human or animal, reference, cell source, cartilage depth, if cells were isolated from healthy (H) or osteoarthritic (OA) cartilage, material models and chondrocyte parameters. Linear elastic model: *E* is the elastic or Young’s modulus (kPa); viscoelastic model: *E*_0_ is the instantaneous Young’s modulus (kPa), *E*_∞_ is the equilibrium Young’s modulus (kPa) and *µ* is the apparent viscosity (kPa s); porohyperelastic model, viscohyperelastic model and poroviscohyperelastic model: *E* is the equilibrium elastic modulus (kPa), *C*_1_ (kPa) and *D*_1_ (kPa^−1^) are the temperature-dependent material constants, *g*_1_ is the Prony shear relaxation (−), *k*_1_ is the Prony bulk relaxation (−), *τ*_1_ is the relaxation time parameter (s), *k* is the hydraulic permeability (m^4^/N s) and *H_A_* is the aggregate modulus (kPa). N/A = not available. Parameter values are reported as mean ± SD, except for those reported by [38] (only values attained via an optimisation procedure).

**Table 3:** Summary table of the mechanical parameters from the analysed literature concerning chondrons.

Mechanical Test	Reference	Cell Source	Cartilage Depth	Material Model	Parameters
AFM ^1^	[25]	Murine spheno-occipital synchondrosis	full thickness	Linear Elastic	*E* = 265 ± 53
	[66] ^4^	Porcine medial condyles	superficial	Linear Elastic	*E* = 54.9 ± 4.5
			middle		*E* = 49.4 ± 4.5
			deep		*E* = 50.6 ± 4.5
	[59]	Human femoral condyles	full thickness	Linear Elastic	*E* = 306 ± 133
		Porcine medial condyles			*E* = 81 ± 19
		Murine knee joint			*E* = 197 ± 92
	[27]	Human femoral medial condyle	full thickness	Linear Elastic	*E* = 137 ± 22
	[67] ^5^	Bovine femoral condyles	cultured	Linear Elastic	*E* = 4.14 ± 0.4
MPA ^2^	[58]	Human femoral head	superficial	Linear Elastic	*E* ^6^ = 68.9 ± 18.9
			middle/deep		*E* ^6^ = 62.0 ± 30.5
			full thickness		*E* ^6^ = 66.5 ± 23.3
					*E* ^7^ = 43.1 ± 17.9
	[68]	Human femoral head	superficial	Biphasic	*E* = 39.7 ± 13.9 *k* = 4.71 ± 4.18
			middle/deep		*E* = 36.8 ± 20.6 *k* = 3.69 ± 3.4
	[60]	Canine femoral condyles	superficial	Linear Elastic	*E* ^6^ = 24.0 ± 10.9
					*E* ^8^ = 25.1 ± 11.5
					*E* ^7^ = 10.8 ± 4.3
			middle/deep		*E* ^6^ = 23.2 ± 7.1
					*E* ^8^ = 23.6 ± 7.3
					*E* ^7^ = 12.1 ± 3.9
Cytomanipulation ^3^	[49]	Bovine trochlear humerus	full thickness	Linear Elastic	*E* = 19 ± 2
				Viscoelastic	*E*_0_ = 27 ± 4 *E*_∞_ = 12 ± 1 *µ* = 3.4 ± 0.6

For each analysed study, the following information is reported: reference, cell source, cartilage depth, material models and chondrocyte parameters. Linear elastic model: *E* is the elastic or Young’s modulus (kPa); viscoelastic model: *E*_0_ is the instantaneous Young’s modulus (kPa), *E*_∞_ is the equilibrium Young’s modulus (kPa) and *µ* is the apparent viscosity (kPa s); biphasic model: *E* is the equilibrium elastic modulus (kPa) and *k* is the hydraulic permeability (m^−13^/N∙s). ^1^ All AFM tests were performed using AFM stiffness mapping and thus without the extraction of the chondron from the cartilage samples except for in [67]. ^2^ All MPA tests were performed on mechanically isolated chondrons. ^3^ Chondrons were enzymatically extracted and tested at 0.3 deformation (linear elastic data) and 0.5 deformation (viscoelastic data). ^4^ The data reported in the work show slight orthogonal anisotropy. ^5^ Data of a cultured chondrocyte in vitro after 28 days. ^6^ Layered model used. ^7^ Half-space model. ^8^ Shell model. Parameters are reported as mean ± standard deviation.

**Table 4:** Poisson’s ratio values assumed in different testing experiments.

Mechanical Test	Reference	Material Model	Poisson’s Ratio *ν* (−)
AFM	[35]	Linear elastic, viscoelastic	0.38
	[36]	Linear elastic, viscoelastic	0.5 (parametric studies show that varying *ν* from 0.3–0.5 altered the measured properties by less than 20%)
	[34]	Linear elastic	0.4
	[37]	Viscohyperelastic	0.499 (high strain rate) 0.35 (low strain rate)
	[38]	Viscohyperelastic, porohyperelastic, poroviscohyperelastic	0.25–0.45
MPA	[41]	Viscoelastic	0.5
	[35]	Viscoelastic	0.38
Cytoindentation	[44]	Linear elastic, Viscoelastic	0.5
Modified cytoindentation	[45]	Linear elastic, Viscoelastic	0.5
	[46]	Viscoelastic	0.26
Micromanipulation	[49]	Non-linear elastic, non-linear viscoelastic	0.5

Poisson’s ratio, *ν*, is reported as mean.

**Table 5:** Summary table of the mechanical parameters from the analysed literature concerning chondrons.

Mechanical Test.	Reference	Cell Source	Cartilage Depth	Material Model	Parameters
AFM	[27]	Human femoral condyle	full thickness	Linear Elastic	*E* = 96 ± 16
MPA	[58]	Human femoral head	superficial	Linear Elastic	*E* ^1^ = 39.1 ± 19.6
			middle/deep		*E* ^1^ = 43.9 ± 23.0
			full thickness		*E* ^2^ = 41.8 ± 21.3
					*E* ^3^ = 33.1 ± 22.9
	[68]	Human femoral head	superficial	Biphasic	*E* = 20.8 ± 19.5 *k* = 10.46 ± 6.96
			middle/deep		*E* = 24.4 ± 12.7 *k* = 9.91 ± 11.3

The studies were divided according to the mechanical test used. All AFM tests used stiffness mapping and thus did not need an extraction method. The MPA tests were all performed on mechanically isolated chondrons. The wide range of the *E* is due to the progressive loss of the mechanical response over time as the disease settles. ^1^ Layered model used. ^2^ Shell model. ^3^ Half-space model. Linear elastic model: *E* is the elastic or Young’s modulus (kPa); biphasic model: *E* is the equilibrium elastic modulus (kPa) and *k* is the hydraulic permeability (m^−13^/N∙s).

## Data Availability

All the results and data have been collected from the reported literature.
